# The prevalence of motility-related genes within the human oral microbiota

**DOI:** 10.1128/spectrum.01264-24

**Published:** 2024-12-09

**Authors:** Sofia T. Rocha, Dhara D. Shah, Qiyun Zhu, Abhishek Shrivastava

**Affiliations:** 1 Biodesign Institute, Arizona State University, Tempe, Arizona, USA; 2 School of Life Sciences, Arizona State University, Tempe, Arizona, USA; 3 School of Mathematical and Natural Sciences, Arizona State University, Glendale, Arizona, USA; Institute of Parasitology, Ceske Budejovice, Czechia

**Keywords:** bacterial motility, gliding motility, type 9 secretion system, flagella, type IV pilus, oral microbiota

## Abstract

The human oral and nasal microbiota contains approximately 770 cultivable bacterial species. More than 2,000 genome sequences of these bacteria can be found in the expanded Human Oral Microbiome Database (eHOMD). We developed HOMDscrape, a freely available Python software tool to programmatically retrieve and process amino acid sequences and sequence identifiers from BLAST results acquired from the eHOMD website. Using the data obtained through HOMDscrape, the phylogeny of proteins involved in bacterial type 9 secretion system (T9SS)-driven gliding motility, flagellar motility, and type IV pilus-driven twitching motility was constructed. A comprehensive phylogenetic analysis was conducted for all components of the rotary T9SS, a machinery responsible for secreting various enzymes, virulence factors, and enabling bacterial gliding motility. Results revealed that the T9SS outer membrane β-barrel protein SprA of human oral bacteria underwent horizontal evolution. Overall, we catalog motile bacteria that inhabit the human oral microbiota and document their evolutionary connections. These results will serve as a guide for further studies exploring the impact of motility on the shaping of the human oral microbiota.

**IMPORTANCE:**

The human oral microbiota has been extensively studied, and many of the isolated bacteria have genome sequences stored on the human oral microbiome database (eHOMD). Spatial distribution and polymicrobial biofilms are observed in the oral microbiota, but little is understood on how they are influenced by motility. To bridge this gap, we developed a software tool to identify motile bacteria from eHOMD. The results enabled the cataloging of motile bacteria present in the oral microbiota but also provided insight into their evolutionary relationships. This information can guide future research to better understand how bacterial motility shapes the human oral microbiota.

## INTRODUCTION

The human oral cavity has different parts that are colonized by bacteria. These include, but are not limited to, teeth, tongue, cheek, nose, and gingival plaques (1). The human oral microbiota is linked to caries (2, 3), tonsillitis (4), gingivitis, periodontitis (5, 6), cardiovascular diseases (7, 8), oral cancer (9, 10), and Alzheimer’s disease (11, 12). Caries occurs when acid-producing bacteria break down dental enamel and dentine, leading to mineral dissolution (3). Bacterial tonsillitis is characterized by inflammation of the tonsils (4, 13), while gingivitis causes red, swollen, and bleeding gums (14). Periodontitis is a more severe form of inflammation that results in gum and bone deterioration and subsequent bone loss. Both gingivitis and periodontitis are associated with the formation of polymicrobial biofilms (5). The oral pathogen *Porphyromonas gingivalis* uses the Type 9 Secretion system (T9SS) to secrete harmful gingipain proteases. These proteases affect the immune system and cause periodontitis (15, 16). Gingipains also have neurotoxic properties and are associated with Alzheimer’s disease (11, 12) and cardiovascular diseases (17–20).

The oral cavity has different gradients influenced by factors like nutrient availability, heat, oxygen, saliva, and crevicular fluid (21). Bacteria that might possess the ability to sense some of these gradients could move toward favorable conditions using chemotaxis. This directed movement may influence the micron-scale spatial distribution of the human oral microbiota. Bacteria employ various motility mechanisms, with flagellar, twitching, and gliding motility being the most notable modes of motility (22–24). Many pathogens and commensals use motility to move to more favorable environments. Here, we catalog motile bacteria found in the human oral cavity that are driven by gliding, flagellar, and twitching motility.

The bacterial T9SS, which is a recently discovered machinery, enables both gliding motility and protein secretion. It utilizes a molecular rack and pinion machinery for gliding, where the T9SS rotary motor acts as a pinion to move a conveyor belt rack (GldJ) on the bacterial cell surface. This system facilitates the movement of adhesins along the cell surface (23, 25, 26). Motile T9SS containing human oral bacteria carry other oral microbes and bacteriophages as cargo (27). A functional T9SS allows for the secretion of over 200,000 bacterial proteins in members of the Bacteroidetes-Fibrobacteres-Chlorobi superphylum (23). Flagellated bacteria have a rotating flagellar motor that is connected via a hook to the flagella. Regulated by the chemotaxis pathway, the flagellar motor enables the bacteria to swim in a specific direction (run). Disruption in the direction of rotation of the flagellar motor results in a tumble. The probability of running or tumbling depends on the chemotaxis gradient (24). Twitching motility is a type of surface-associated movement facilitated by type IV pili. Slender filaments (PilA, PilE) adhere to a solid surface and undergo rapid extension cycles at their ends. The filament retraction, powered by an ATP-driven motor called PilT, pulls the cell forward (28–30).

The Human Oral Microbiome Database (eHOMD) houses over 2,000 genome sequences from human oral and nasal isolates, encompassing 775 bacterial types across 185 genera (31). In this study, an extensive search was conducted on eHOMD to identify proteins involved in the three motility systems described above. Computational tools were developed to automate the retrieval of sequence identifiers and amino acid sequences for the identified proteins. This resulted in a comprehensive catalog of potentially motile bacteria found in the human oral microbiota. A phylogenetic analysis shed light on the evolutionary lineages of these motility-related proteins. Interestingly, the analysis of the T9SS machinery, which is relatively understudied, revealed evidence of horizontal gene transfer in the outer-membrane pore protein and regions of structural similarities between T9SS rotor and conveyor belt proteins.

## RESULTS

### Development and use of HOMDscrape for automated generation of a curated data set from BLAST results

eHOMD is an online database that contains the genome sequences of bacteria found in the human oral and nasal microbiome. The online database utilizes SequenceServer (2.0.0) (32) to generate a BLAST database custom to eHOMD. The version 2 of eHOMD releases all their BLAST results with a sequence identifier and does not have a user-friendly function that allows a user to save sequence identifiers along with the species name and amino acid sequence. To automatically create a database that can easily be used for downstream analysis, we developed a local software named HOMDscrape to programmatically retrieve and process eHOMD search results.

**Fig 1:**
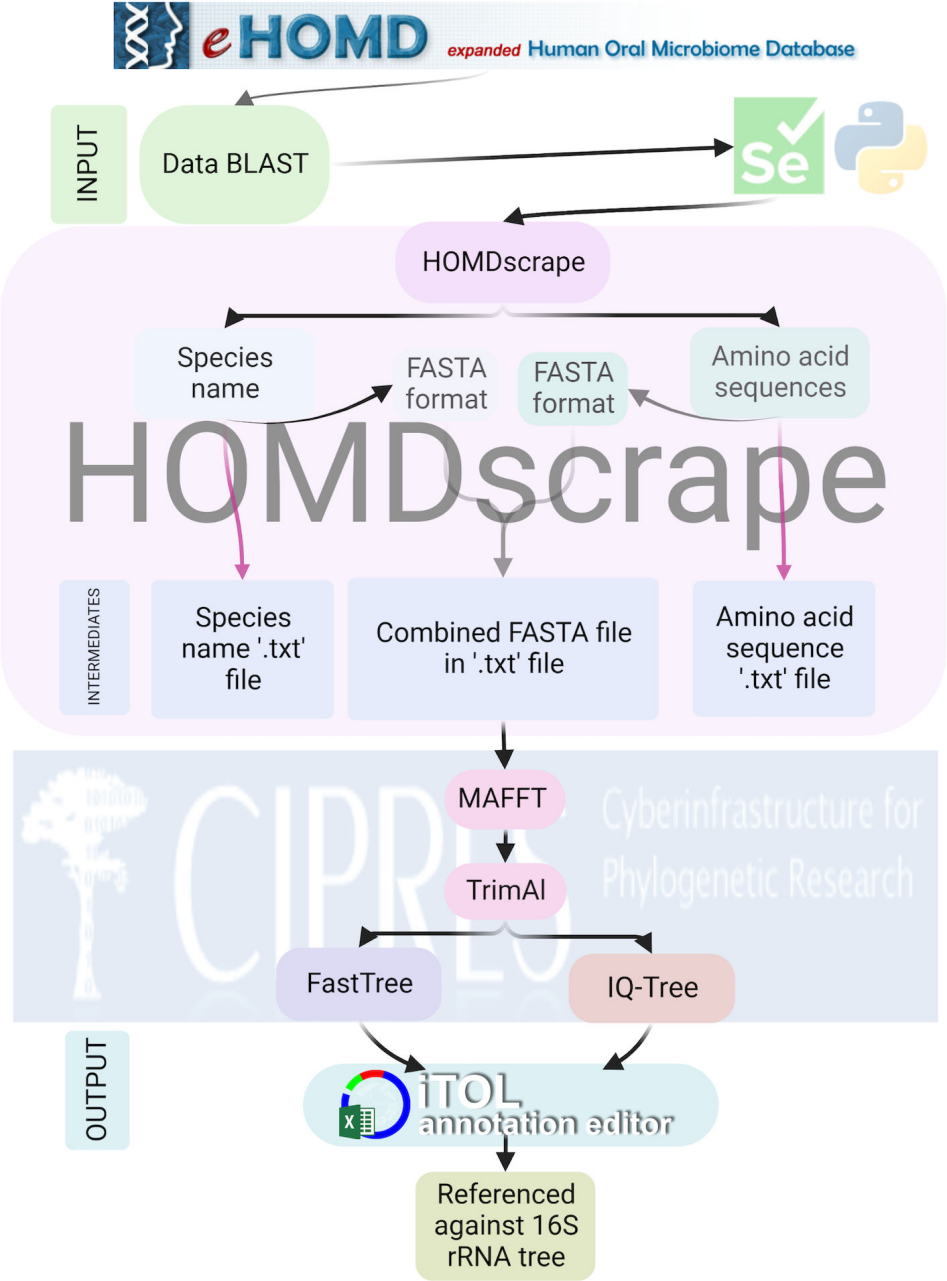
An outline of HOMDscrape, a selenium Python software that extracts bacterial species names and amino acid sequences from BLAST results on eHOMD. Black arrows are associated with the main files used for analysis, while pink arrows indicate additional intermediate files.

HOMDscape is a Python-based software tool freely available on GitHub (https://github.com/strocha1/HOMDscrape). It utilizes the chromedriver library (version 96.0.4664.45) to automate interaction with the eHOMD webserver using an open-source API and the HTML code from eHOMD. It is used to automate the process of gathering species names and amino acid sequences from the BLAST result. A schematic depicting each step of HOMDscrape is outlined in Fig. 1. Once the raw data is collected by HOMDscrape, it is converted into a FASTA format and saved as a plain text file that can be used for downstream analysis.

### A phylogenetic tree of human oral bacteria cataloged on eHOMD

The phylogenetic tree of eHOMD-hosted genomes was reconstructed using their 16S rRNA gene sequences (Fig. S1). This tree illustrates the evolution of all the species described on the eHOMD. The 16S rRNA RefSeq data available on eHOMD was used as raw data for phylogenetic reconstruction. The trees constructed later in this study, with the data collected by HOMDscrape, were compared with this 16S tree. Except for this 16S rRNA analysis, all phylogenetic analyses were conducted using the data obtained and parsed by HOMDscrape. The branches are colored corresponding to the bootstrap value. The colormap indicates bootstrap values. Branch lengths are incorporated into the size of the tree and denoted by a tree scale. The maximum branch length was 4.71 from tip to root, noted as the branch length baseline.

### Prevalence of T9SS in the human oral microbiota

**Fig 2:**
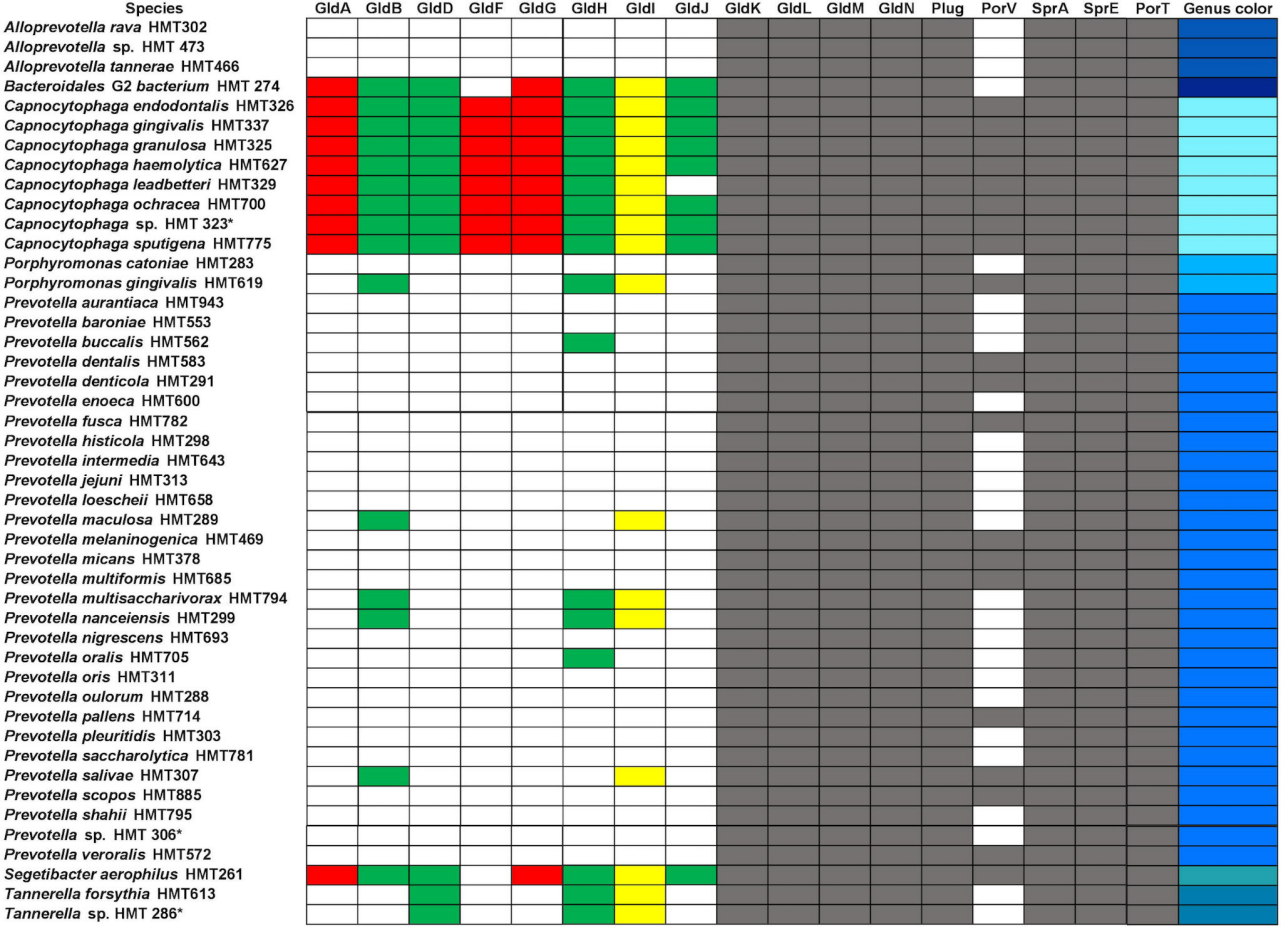
Summary of the genera containing type 9 gliding motility proteins. Species marked with a “*” represent unnamed isolates. A comprehensive list with species names is available in Table S2. Colored cells refer the presence of the protein, while a white cell refers to the absence of the protein. A gray color refers to core T9SS proteins, while proteins labeled with red are involved with ABC transporters, green are additional proteins required for gliding motility, and yellow are peptidylprolyl isomerase proteins. The genus color column corresponds to the colormap used in T9SS phylogenetic trees.

T9SS has several structural proteins that are necessary for both secretion and motility, while some proteins are only involved in motility (23, 33). T9SS is a recently discovered machinery and very little is known about its evolution. To fill this knowledge gap, we searched for 16 major T9SS proteins and analyzed their evolutionary patterns. While all 16 proteins are required for motility, only a fraction of them are required for the T9SS to secrete proteins for nonmotile organisms (23). We predict that the major proteins forming the T9SS are present in 68 species across 7 genera isolated from the human oral microbiota. However, 20 of these species may exhibit gliding motility due to the presence of essential motility proteins. (Fig. 2; Table S2).

**Fig 3:**
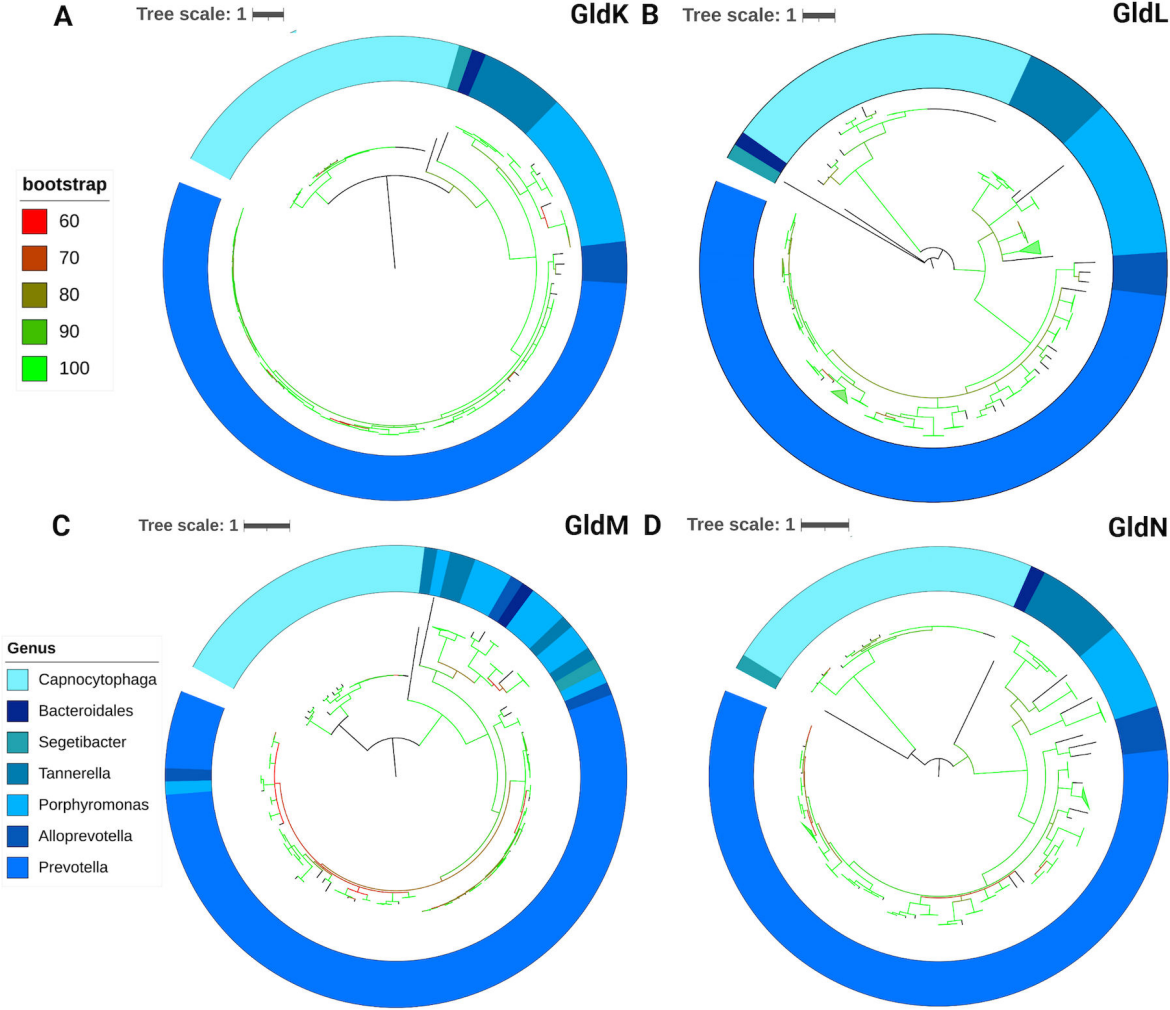
(**A–D**) Phylogenetic trees of GldK, GldL, GldM, and GldN where the colormap is depicted via the column “Genus color” in Fig. 2. The color of tree branches indicates bootstrap values.

The predicted rotary component of T9SS is made up of four proteins. GldK/PorK, GldL/PorL, GldM/PorM, and GldN/PorN. GldK and GldN form a periplasmic ring, while GldL and GldM form a transmembrane ion channel that powers T9SS rotation (23, 34, 35). GldL and GldM are structurally similar to the 5:2 MotA-MotB complex found in the flagellar motor. However, there is no sequence similarity between GldL-GldM and MotA-MotB (35). Using HOMDscrape, we found that 71 species of oral bacteria that belong to seven genera contained GldK. Additionally, 70 species within the same seven genera had GldL, while 71 species had GldM, and 68 species had GldN (Fig. 3). Their maximum branch lengths from tip to root were 4.21 for GldK, 9.38 for GldL, 3.13 for GldM, and 3.32 for GldN, suggesting that GldK, GldM, and GldN diverged at a similar rate to the 16S tree, while GldL had more evolutionary changes.

PorT (SprT) is an outer membrane protein that is required for the secretion of gingipains in *P. gingivalis* (23, 36, 37). We found that 71 oral microbial species within 7 genera had PorT (Fig. S2). SprE (PorW) is required for the secretion of a variety of T9SS proteins and is predicted to act as a bridge between the SprA translocon and GldK/N rings (23, 34). We found that 92 oral microbial species within 19 genera had SprE (Fig. 2; Fig. S2). The maximum branch length values from tip to root were 4.27 for PorT and 5.60 for SprE, diverging at a similar rate to that of the 16S tree. In summary, our analysis showed that seven different genera of eHOMD cataloged bacteria, namely, *Capnocytophaga, Porphyromonas*, *Prevotella, Bacteroidales [G-2], Tannerella, Segetibacter,* and *Alloprevotella* possess the core components of T9SS (Fig. 2).

### Horizontal evolution of the T9SS translocon

**Fig 4:**
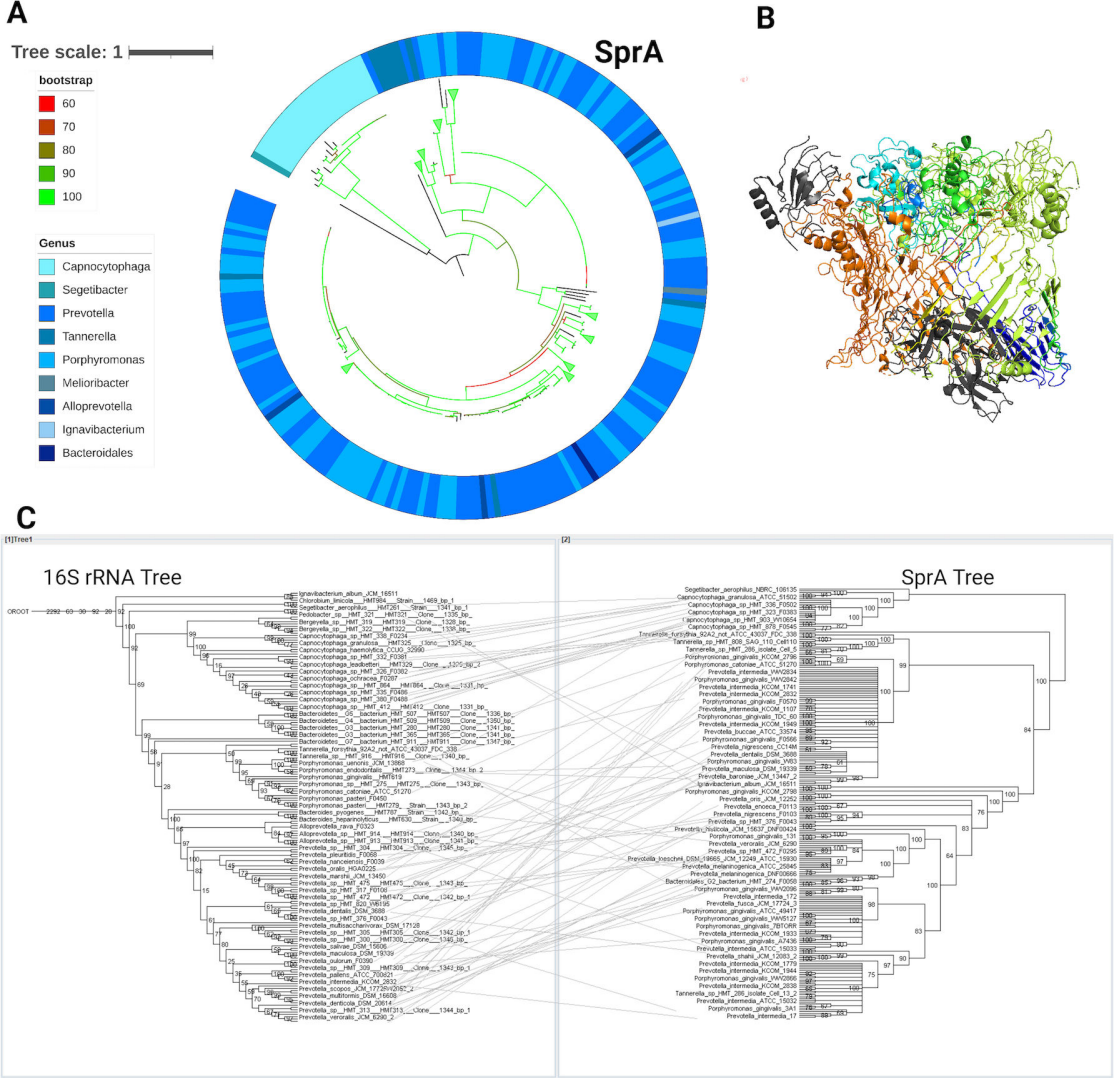
(**A**) Phylogenetic tree of SprA where the colormap is depicted via the column “Genus color” in Fig. 2. The color of tree branches indicates bootstrap values. (**B**) Structure of SprA where domains are labeled with separate colors. (**C**) Tanglegram of 16S rRNA tree (Left) and SprA (Right).

SprA is the largest single polypeptide outer membrane β-barrel that acts as the protein translocon of T9SS (23, 38). SprA interacts with PorV and the T9SS Plug to form an outer membrane gate (38). We found that 73 oral microbial species within nine genera had SprA (Fig. 4). Forty-one oral microbial species within 9 genera had PorV, and 72 oral microbial species within 9 genera had the T9SS Plug. The maximum branch length values of the phylogenetic trees from tip to root were 2.11 for SprA, 2.47 for PorV, and 3.42 for Plug, suggesting that there were fewer evolutionary changes than that of the 16S tree. Members of the *Melioribacter* and *Ignavibacterium* genera contain the PorV, SprA, and Plug complex but does not contain any other T9SS protein (Fig. 4; Fig. S3).

The phylogenetic tree of SprA had a higher number of inconsistent terminal species when compared to the phylogenetic tree of the 16S rRNA gene (Fig. 4). The terminal nodes were inconsistent after the *Capnocytophaga* genus. This suggests that there was a higher level of recombination, and there is a possibility that the SprA protein is not well conserved, causing the deviation from the 16S rRNA phylogenetic tree. SprA is a 267 KDa polypeptide and has roughly 7 domains (38). In order to determine if the deviation from the 16S rRNA tree is found throughout the whole protein or is limited to certain domains, a series of tanglegrams were constructed for each SprA domain (Fig. S4 to S10) (39). While there was deviation from the 16S rRNA tree across all domains, a lower rate of deviation was found in domains 2, 5, and 7 as compared with the other four domains (Fig. S5, S8, and S10). This suggests that domains 2, 5, and 7 may be under stronger selective pressure to maintain their function, while other domains might be experiencing more recombination or diversification. This could imply that evolutionary changes in SprA are driven by both domain-specific functional requirements and broader evolutionary pressures.

### Prevalence and phylogeny of the ABC transporter domain containing gliding proteins

GldA is essential for gliding motility, and it is predicted to be an ABC transporter that interacts with T9SS. GldG is predicted to be a transmembrane protein that interacts with GldF and GldA to form an ABC transporter. GldF is essential for gliding motility and is required for the stability of GldK, whereas GldB and GldD are lipoproteins that are essential for gliding motility (40, 41). Using HOMDscrape, 22 oral microbial species in three genera were discovered to contain GldA. In parallel, 29 oral microbial species in 7 genera had GldB and 25 oral microbial species in 4 genera contained GldD and 22 oral microbial species in 3 genera had GldF (Fig. S11). Similarly, 22 oral microbial species in 3 genera had GldG (Fig. S12). The maximum branch length values for GldA, GldB, GldD, GldF, and GldG were 1.39, 2.15, 2.77, 2.26, and 2.20, suggesting that there were fewer evolutionary changes than that of the 16S tree.

### Phylogeny of proteins that either form or stabilize the mobile cell-surface track driven by T9SS

**Fig 5:**
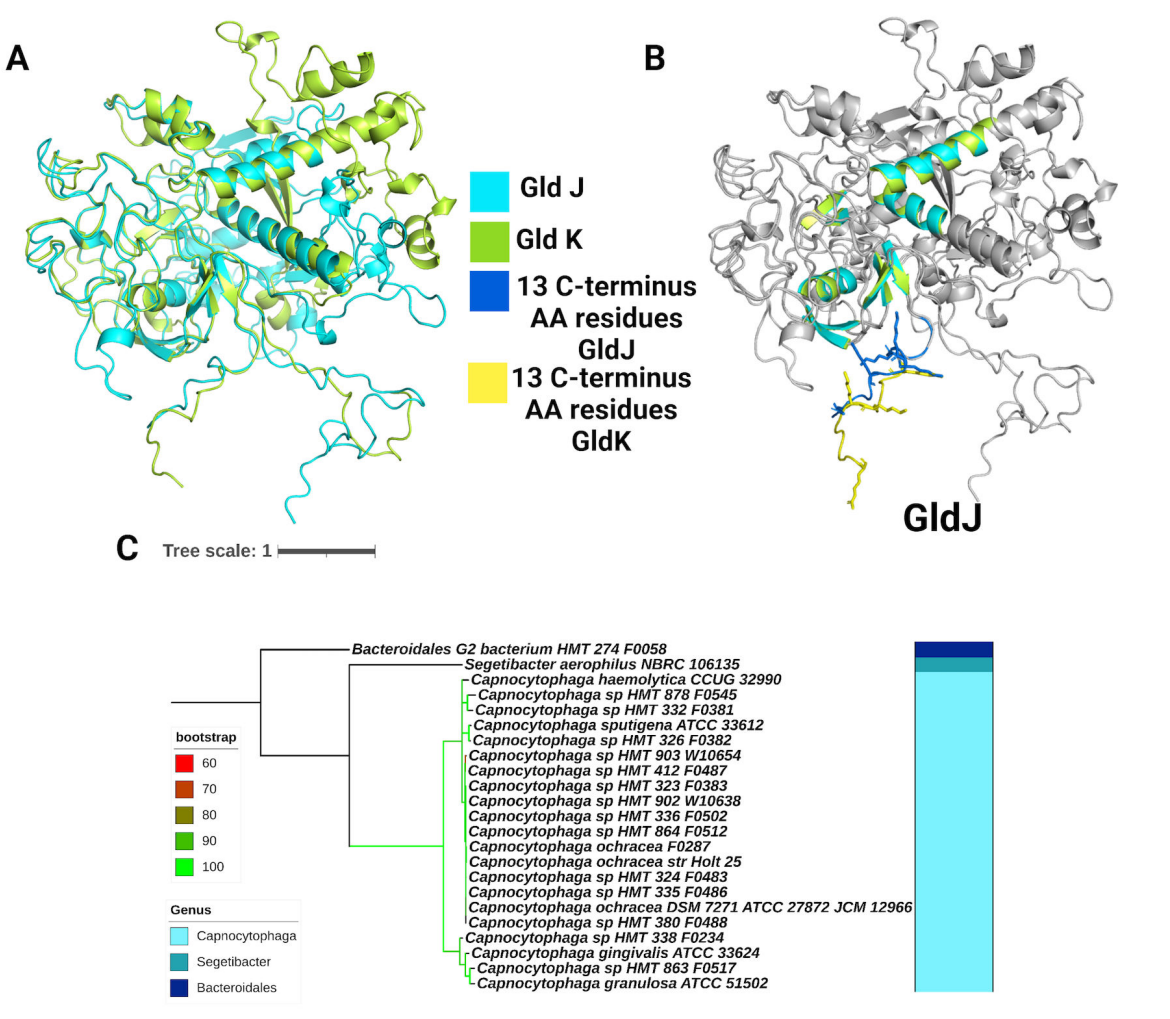
(**A**) Superimposed structures of GldJ (blue) and GldK (green). (**B**) Superimposed structure from A, with only highlighted regions of similarity. (**C**) Phylogenetic tree of GldJ. The color of tree branches indicates bootstrap values.

GldH and GldI are essential for gliding motility and help stabilize GldJ (23, 33, 42). Thirty-nine oral microbial species in 8 genera had GldH. Thirty-two oral microbial species in 8 genera had GldI. However, only 21 oral microbial species in 3 genera had GldJ (Fig. 5D; Fig. S12). The maximum branch length values for GldH, GldI, and GldJ were 4.16, 2.15, and 2.21, where GldH evolves similar to that of the 16S tree and GldI and GldJ have fewer evolutionary changes.

GldJ forms helical tracks on the cell surface that acts as the rack in the rack and pinion assembly that drives gliding motility (40, 43). When comparing the phylogenetic tree of the T9SS gliding protein GldJ to other core T9SS proteins, a decrease in genera containing GldJ is observed. Surprisingly, bacteria containing GldJ constitute around 1% of the total diversity of the human oral microbiota but constitute 10% of total microbial abundance at important gingival sites within the human oral cavity (31, 44, 45).

GldJ of the motile *Capnocytophaga gingivalis* has 31% amino acid sequence similar to the T9SS ring protein GldK of *C. gingivalis*. GldJ and GldK are closely related lipoproteins that exhibit sequence similarity to sulfatase-modifying enzymes such as formylglycine-generating enzyme (FGE) but lack certain active site residues of FGE (46). The predicted Alphafold (47) structures of GldJ and GldK were generated (Fig. 5), and the confidence in predictions per residue through the local distance difference test score (lDDT) (48) was plotted against residue positions (Fig. S13). Aligned GldJ and GldK of *C. gingivalis* show RMSD of 0.58 Å for the total of 1,371 atoms. At the N-terminus of both proteins, beta sheets consisting of residues 39–42 of GldJ and 25–28 of GldK, 45–49 of GldJ and 31–35 of GldK, 65–69 of GldJ and 51–55 of GldK superimposed completely. Additionally, alpha helices at the N-terminus with residues spanning 81–94 of GldJ and 67–82 of GldK were also well aligned in both structures. In the middle part of the protein, residues 148–158 of GldJ and 251–261 of GldK, and residues 238–246 of GldJ and 285–293 of GldK that form alpha helices, show significant alignment. At the C-terminus of both proteins, there are three beta sheets that are well aligned. These are residues 487–490 of GldJ and 384–387 of GldK, residues 508–511 of GldJ and 405–408 of GldK, and residues 523–526 of GldJ and 420–423 of GldK (Fig. 5). All residues are numbered after removal of the signal peptides at the N-terminus of both GldJ and GldK of *C. gingivalis*. GldJ of an environmental isolate *Flavobacterium johnsoniae* is required for the stability of T9SS, that is, GldK is not detected in mutants that lack the full-length GldJ. However, when only 8–13 amino acid containing C-terminus of GldJ is deleted, secretion by T9SS occurs but motility is significantly diminished. The C-terminus of GldJ forms an electrostatically rich region, and it appears to play a crucial role in enabling gliding motility (27). The C-terminus of GldK of *C. gingivalis* also appears to be electrostatically rich but Arginine rather than Lysine is more prevalent.

### Prevalence of flagellar motility in the human oral microbiota

The flagellar motor and the virulence-associated type III injectosome share several structural proteins (49, 50), and both systems have the type III secretion system for protein export. To reduce false positives during our bioinformatics search for flagellar motility, it was important to exclude flagellar proteins that are found in the type III secretion system or the type III injectosome. We compared the proteins that form the flagellar motor and the type III injectosome (Table S1). This comparison led to the selection of three proteins, FliC, FlgK, and FlgL as markers for the presence of flagellar motility that are absent in the straight-shaped injectisome. FliC is the main structural subunit in the flagellar filament, while FlgK and FlgL form the hook-filament junction (49, 51).

**Fig 6:**
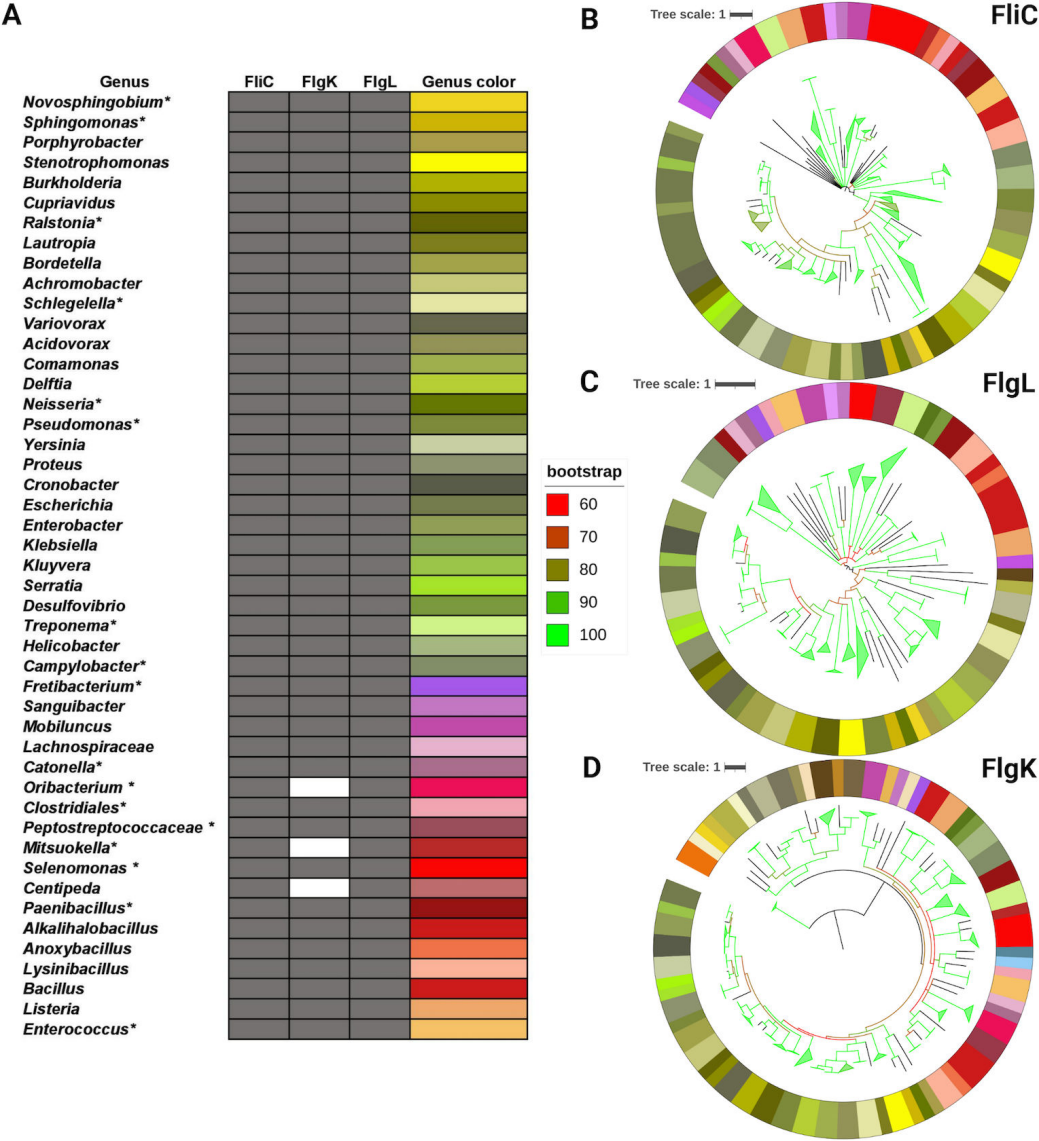
(**A**) Summary of the genera containing flagellar motility proteins. Genera marked with “*” display differences at the species level. A comprehensive list with species names is available in Table S2. (**B–D**) Phylogenetic trees of FliC, FlgL, and FlgK. The colormap for Trees is depicted via the column “Genus color” in Fig. 6A. The color of tree branches indicates bootstrap values.

We predict that 47 genera and 78 species in the human oral microbiota have flagellar motility (52) (Fig. 6; Table S2). We identified FliC in 84 species of 48 genera, FlgK in 108 species of 64 genera, and FlgL in 75 species of 49 genera (Fig. 6; Table S2). The evolutionary pattern of FliC, FlgK, and FlgL in human oral bacteria displays similarities to the terminal nodes present in the 16S rRNA tree (Fig. 6; Fig. S1), and several genera were predicted to contain the genes necessary for motility across all species. The inconsistencies in motility gene predictions between species are marked with an Asterix (Fig. 6). For all phylogenetic trees presented in this study, the periphery of the tree is labelled by a colored circle that represents the genus corresponding to the terminal node (53). The branches are colored corresponding to the bootstrap value. The colormap indicates bootstrap values. Branch lengths are incorporated into the size of the tree and denoted by a tree scale. The maximum branch length for FliC, FlgK, FlgL were 6.18, 5.07, and 3.48, respectively. This trend seems to mostly follow the 16S rRNA tree which has a maximum branch length of 4.71.

### Prevalence of type IV pilus-driven twitching motility in the human oral microbiota

**Fig 7:**
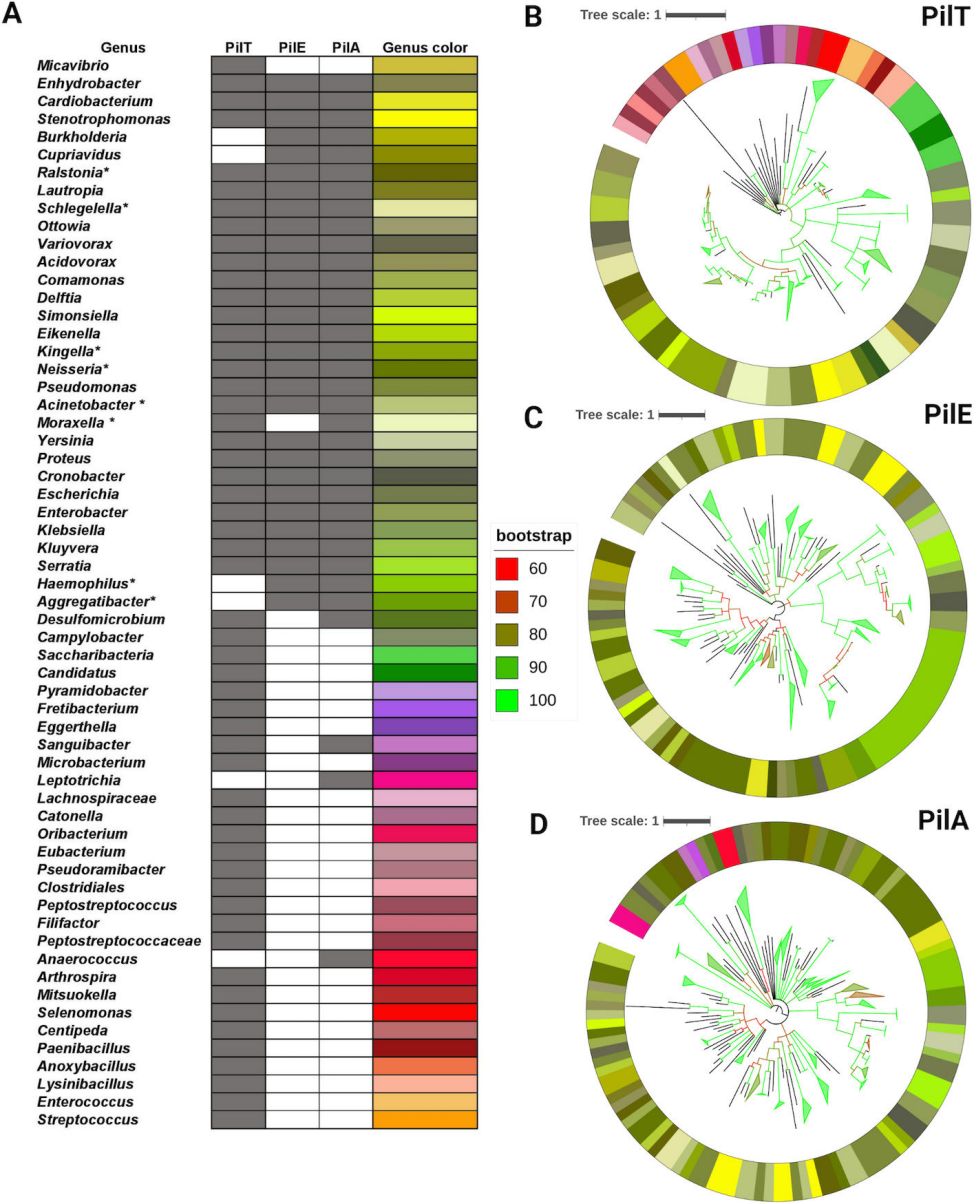
(**A**) Summary of the genera containing type IV pili twitching motility proteins. Genera marked with “*” display differences at the species level. A comprehensive list with species names is available in Table S2. (**B–D**) Phylogenetic trees of PilE, PilT, and PilA. The colormap for Trees is depicted via the column “Genus color” in Fig. 7A. The color of tree branches indicates bootstrap values.

The type IV pilus motor shares several homologous proteins with the type 2 secretion system (T2SS) (28, 54). Proteins that were found in both type IV pilus machinery and the T2SS were excluded from the downstream analysis, and three structural proteins, PilT, PilE, and PilA, were used as markers for the presence of type IV pilus-driven twitching motility. PilT functions as the retraction ATPase that aids in twitching motility. PilE functions as the major pilin in bacteria such as *Neisseria gonorrhoeae* and a minor pilin in *Pseudomonas aeruginosa,* while PilA functions as a major pilin in *P. aeruginosa* (28, 55). We predict that 32 genera made up of 58 species have the ability to perform twitching motility (Fig. 7; Table S2). PilT is found in 105 species of 56 genera, making up to 30% of the total genera found in the human oral microbiota. In addition, 76 species of 30 genera had PilE and 85 species of 35 genera had PilA (Fig. 7; Table S3). The evolutionary pattern of PilT, PilE, and PilA in human oral bacteria displays similarities to the 16S rRNA tree (Fig. 7; Fig. S1). Several genera were predicted to contain the genes necessary for motility across all species; inconsistences in motility gene predictions between species have been denoted with an Asterix (Fig. 7). Branch lengths are incorporated into the size of the tree and denoted by a tree scale. The maximum length of the tree from tip to root for PilT, PilE, and PilA were 2.47, 2.96, and 3.07, respectively. This suggests that there is less genetic change between twitching motility proteins as compared to the 16S rRNA tree which indicates that there is less evolutionary distance or genetic variation among these proteins across species.

## DISCUSSION

In agreement with evolutionary trends, the terminal species on the phylogenetic trees of the motility proteins analyzed in this study were expected to match the terminal species of the phylogenetic tree of the 16S rRNA gene (56–58). However, the evolution of T9SS protein SprA was an exception to this trend. In the phylogenetic tree of SprA, there was a relatively higher number of inconsistent terminal species when compared to the phylogenetic tree of the 16S rRNA gene or other T9SS gene trees. This suggests that horizontal transfer, followed by genetic drift, might occur in the case of SprA. (Fig. 4). Changes in the confirmation of outer membrane beta barrels can lead to diversification of their functions (59). While the mechanism for SprA’s jump across lineages remains unknown, it is possible that horizontal gene transfer could lead to different conformations of the beta barrel of SprA. Additionally, the phylogenetic trees for the T9SS protein component GldM, type IV Twitching motility protein PilA, and flagellar motility FlgK exhibited minor inconsistencies from the 16S rRNA tree, suggesting that the proteins are exhibiting a slight genetic variation compared to the 16S rRNA tree. Overall, there is a smaller evolutionary distance between the proteins across species, implying that the proteins are subject to stronger evolutionary constraints. This highlights the importance of motility in the human oral microbiota.

Inconsistencies in motility predictions were found at a species level for both flagellar and twitching motilities and were noted with an Asterix (Fig. 6 and 7). This can be due to the quality of sequences displayed on eHOMD or due to loss of functions from mutations or evolutionary pressures such as nutrient availability or competition. An example of this are Campylobacter species where they can be motile via a polar flagellum (*Campylobacter sputorum*) but also contain nonmotile species (*Campylobacter gracilis*). Another example of this can be seen in Entercoccus species where they can be motile via flagella (*Enterococcus casseliflavus*) but is mostly made up of nonmotile species (*Enterococcus faecalis, Enterococcus durans*).

T9SS is found in bacteria of the Fibrobactertes-Bacteroidetes-Chlorobi superphylum. Several members of the Bacteroidetes phylum play important roles in health, and changes in their abundance correlate with diseases such as diabetes, obesity, and ulcerative colitis (60–63). Bacteroidetes in the human oral microbiota employ T9SS for diverse purposes. For example, *P. gingivalis* secretes immunomodulatory gingipain proteases through the T9SS, while *Tannerella forsythia* uses the T9SS to transport virulence-associated cargo proteins to their cell surfaces (64). In motile Bacteroides, such as *Capnocytophaga ochracea*, the T9SS enables gliding motility and biofilm formation (65). T9SS-driven gliding bacteria such as *Capnocytophaga gingivalis* transport other non-motile oral microbial species as cargo, and they play a potential role in shaping the biogeography of the human oral microbiota (27). The analysis presented here shows that although around 4% of the genera found in the human oral microbiota encode the T9SS (Fig. 3), they constitute about 15% of total microbial abundance at important gingival sites within the human oral cavity (44, 45). The T9SS enhances adhesion to surfaces and other microorganisms, which could potentially aid in the improvement of colonization. Additionally, certain enzymes secreted by the rotary T9SS have the capability to break down complex polysaccharides and human tissue, making them a potential source of nutrients for a diverse microbial community. Within the oral microbiota, these characteristics may contribute to the proliferation of microorganisms that utilize T9SS for both nutrient degradation and gliding motility.

Motility is vital for a wide variety of functions which enables movement toward nutrient resources or away from toxic substances, the dispersal of progeny, and the ability to translocate to preferred hosts. These capabilites are evident in various environments such as the the mammalian gastrointestinal tract where *Helicobacter pylori* uses motility to navigate and colonize its preferred niche (66). In the ocean, *Vibrio fischeri* utilizes motility to translocate to their preferred host, the *Euprymna scolopes* squid (67). *Pseudomonas aeruginosa,* which can cause disease in plants, animals, and humans, use motility for adhesion and biofilm formation (68). The freely available HOMDscrape tool was created to build a database of motile microorganisms found in the human oral microbiota. HOMDscrape is a versatile tool that proves valuable not only in the study of motility but could also aid in the analysis of evolutionary patterns within any system encoded by the human oral microbiota. In addition to a detailed analysis of the evolution of T9SS and gliding motility, we predict that around 25% of the genera found in the human oral microbiota have flagellar motility. Additionally, 30% of the genera found in the human oral microbiota encoded the proteins necessary for type IV pilus-driven motility. We find that the ability to move is present in several commensals and pathogenic bacterial species of the human oral microbiota (Fig. 6 and 7; Table S2). Our predictions pave the way for several testable hypotheses that can be experimentally verified in the future. Overall, the catalog of motile human oral bacteria described in this study could serve as a database for future research that explores how motility might shape the human oral microbiota.

## MATERIALS AND METHODS

### Choice and search for genes encoding motility proteins in eHOMD

Amino acid sequences were searched using Basic Local Alignment Search Tool (BLAST) against the genomes hosted at eHOMD. The template sequences were derived from the following model organisms: Flagellar system*—Escherichia coli,* type IV pili system*—Neisseria meningitidis* and *Pseudomonas aeruginosa,* T9SS— *Capnocytophaga gingivalis*. BLAST hits were filtered depending on the protein’s known functions, query sequence similarity, and *E*-values (34, 69). The *E*-values cutoffs used for each protein are described in Fig. S15. New genomes and updated bacterial names can be added with newer versions of HOMD. The aim of this study is to be exhaustive in cataloging of motility proteins in the current version of HOMD. As HOMD gets updated, we call on the community to help update the future iterations of this catalog.

### Multiple sequence alignment

In order to create phylogenetic trees of each motility protein, the following programs were used from the Cyberinfrastructure for Phylogenetic Research computing cluster (CIPRES) (70): MAFFT (Multiple Alignment using Fast Fourier Transform) on Extreme Science and Engineering Discovery Environment (XSEDE) (7.490) (71), TrimAl on XSEDE (1.2.59) (72), FastTreeMP on XSEDE (2.1.10) (73), and IQTree on XSEDE (2.1.2) (53). MAFFT was used to align the multiple sequences gathered by HOMDscrape and was run at the standard parameters set by CIPRES (Fig. S16). MAFFT was performed using the distance metric 6merpair which utilizes a 200PAM/kappa = 2 Nucleic Acid matrix selection and BLOSUM Amino Acid selection. To remove unreliable alignment regions, the result of the MAFFT alignment was run through TrimAl at the standard parameters (Fig. S17).

### Phylogenetic tree reconstruction

IQTree (2.1.2) was used to create maximum likelihood trees. It was run with the standard parameter settings with 10,000 ultrafast bootstrap replicates to generate branch support statistics (Table S4). FastTree (2.1.10) was used to create secondary fast approximate maximum likelihood trees and was run at the standard parameters set by CIPRES (Fig. S18). CIPRES has the function with the IQ-Tree program to allow for an automatic model selection (-m TEST). The constructed trees were then visualized and annotated using iTOL’s online tool and rooted at midpoint (version 6.5.2) (74). A 16S rRNA tree was created using the 16S rRNA RefSeq alignment file (V15.2) available on the HOMD website.

### Tanglegrams for SprA evolution

Tanglegrams were created using Dendroscope v3.8.5 to compare phylogenetic trees of the full SprA and each domain of SprA (39). Species names from the 16S rRNA tree were linked to their respective species name in the phylogenetic trees of SprA and its domains.

### Protein structure alignment

Template sequences for *Capnocytophaga gingivalis* GldJ and GldK were aligned using MAFFT as described above. Structures of GldJ and GldK without signal peptides were generated by Alphafold2 with MMseqs2 (ColabFold v1.5.2) (47, 75). The structures were aligned and visualized using PyMol (76) alignment tool with an align method that uses five iteration cycles and a cutoff of 2 Å. The lipoprotein signal peptide sequence was removed from the beginning of the N-terminal sequences used for GldJ and GldK.

## Data Availability

A version of HOMDscrape is freely available on Github and licensed with an MIT license (https://github.com/strocha1/HOMDscrape). All additional data are available upon request.

## SUPPLEMENTAL MATERIAL

Supplemental material spectrum.01264-24-s0001.pdf Supplemental figures, tables, and text.

Table S1 spectrum.01264-24-s0002.xlsx Comparison table of the flagellar motor and the virulence-associated type III injectosome structural proteins.

Table S2 spectrum.01264-24-s0003.xlsx Summarized motility predictions of species cataloged on eHOMD.

Table S3 spectrum.01264-24-s0004.xlsx Comparison table of type 4 pilus driven twitching motility proteins and type 2 secretion system proteins.

Table S4 spectrum.01264-24-s0005.xlsx Parameters for IQ-Tree.

ASM does not own the copyrights to Supplemental Material that may be linked to, or accessed through, an article. The authors have granted ASM a non-exclusive, world-wide license to publish the Supplemental Material files. Please contact the corresponding author directly for reuse.
