# Supplementary material for: The prevalence of motility-related genes within the human oral microbiota
Source: Microbiol Spectr. 2024 Dec 9;13(1):e01264-24. doi: 10.1128/spectrum.01264-24 (PMC11705866; doi:10.1128/spectrum.01264-24)
Supplement: Supplemental material — Supplemental figures, tables, and text. [file spectrum.01264-24-s0001.pdf]

Supplementary Information

The prevalence of motility within the human oral microbiota

Authors. Sofia T. Rocha, Dhara D. Shah, Qiyun Zhu, Abhishek Shrivastava

Supplementary Figures.

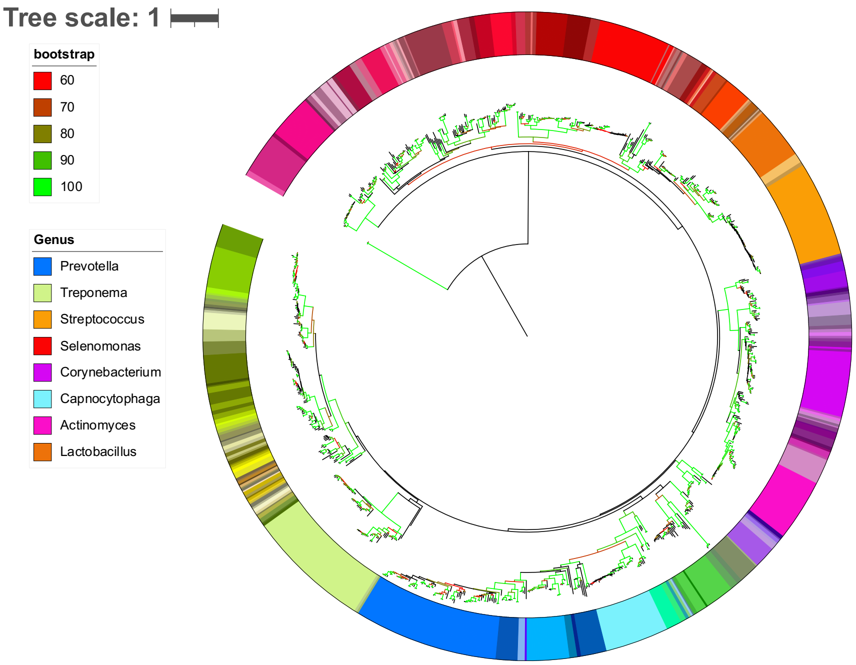

Figure S1. Phylogenetic tree of 16S rRNA RefSeq from eHOMD. An extended colormap for all genera is outlined in Table S2.

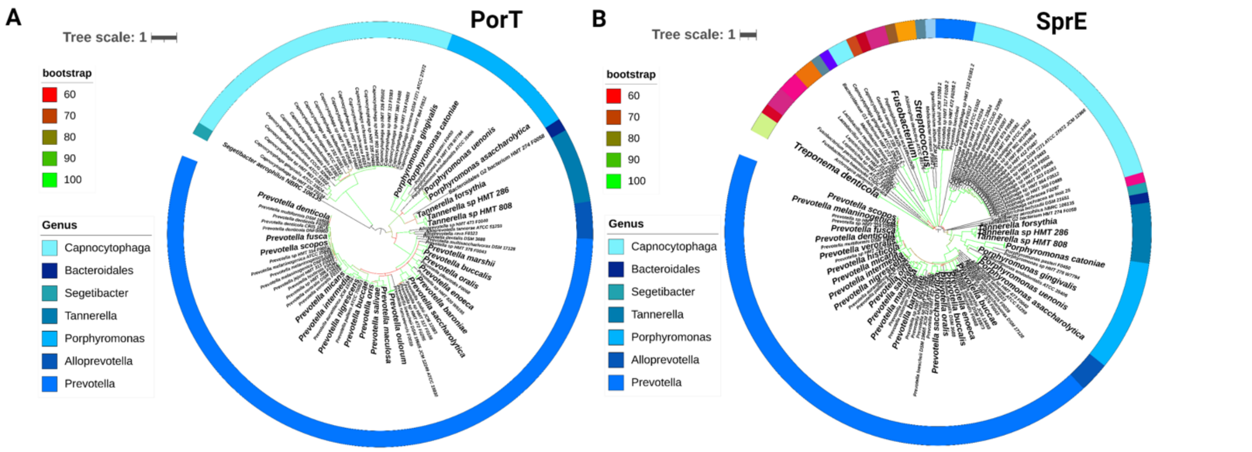

Figure S2. A. Phylogenetic tree of PorT, **B.** Phylogenetic tree of SprE. An extended colormap for all genera is outlined in Table S2.

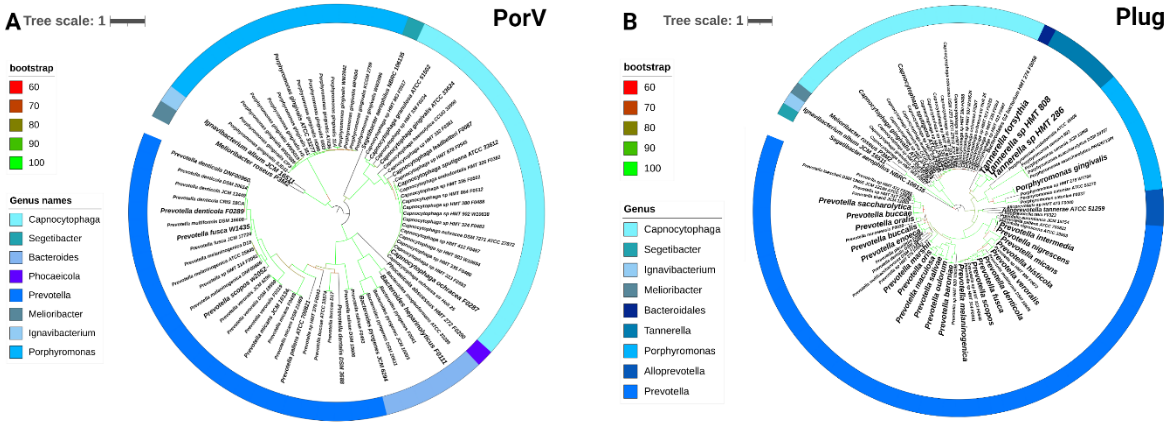

Figure S3. **A.** Phylogenetic tree of PorV, **B.** Phylogenetic tree of the Plug.

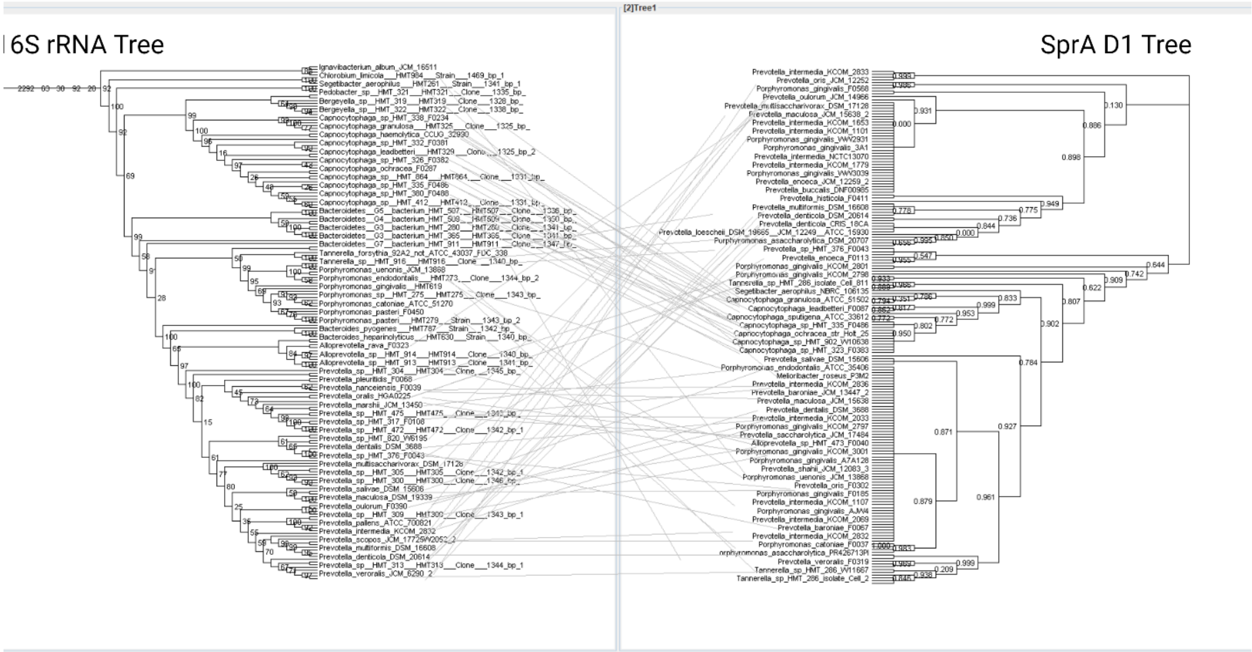

Figure S4. Tanglegram of 16S rRNA tree (Left) and the first domain of SprA (Right)

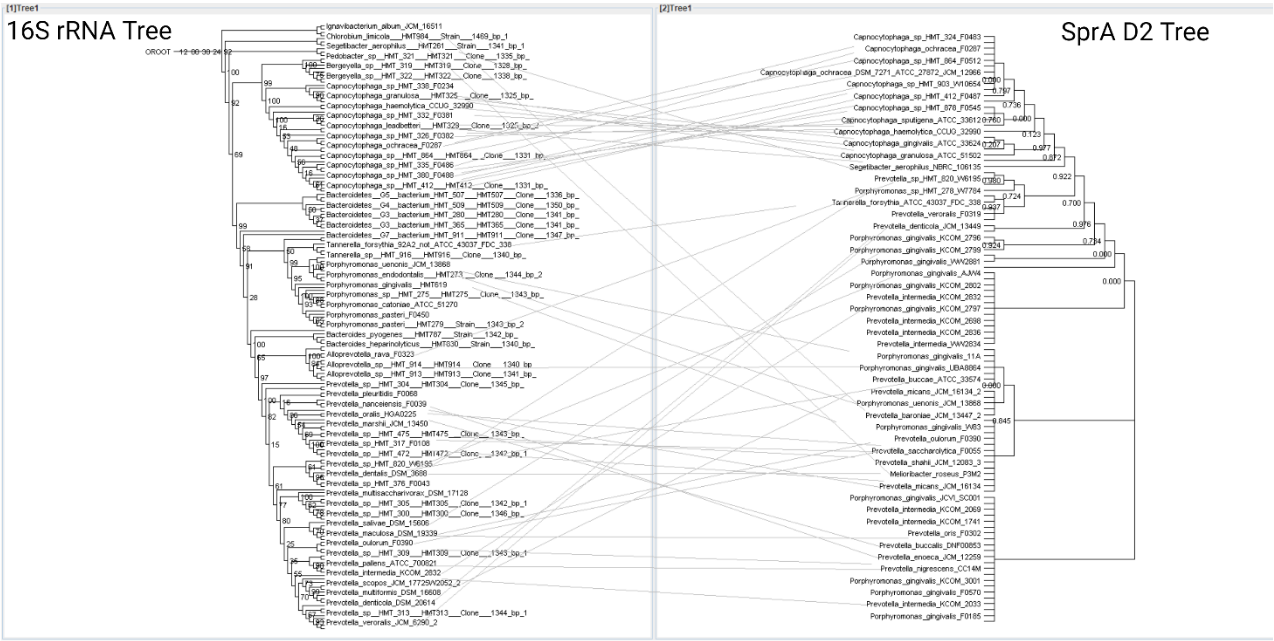

Figure S5. Tanglegram of 16S rRNA tree (Left) and the second domain of SprA (Right)

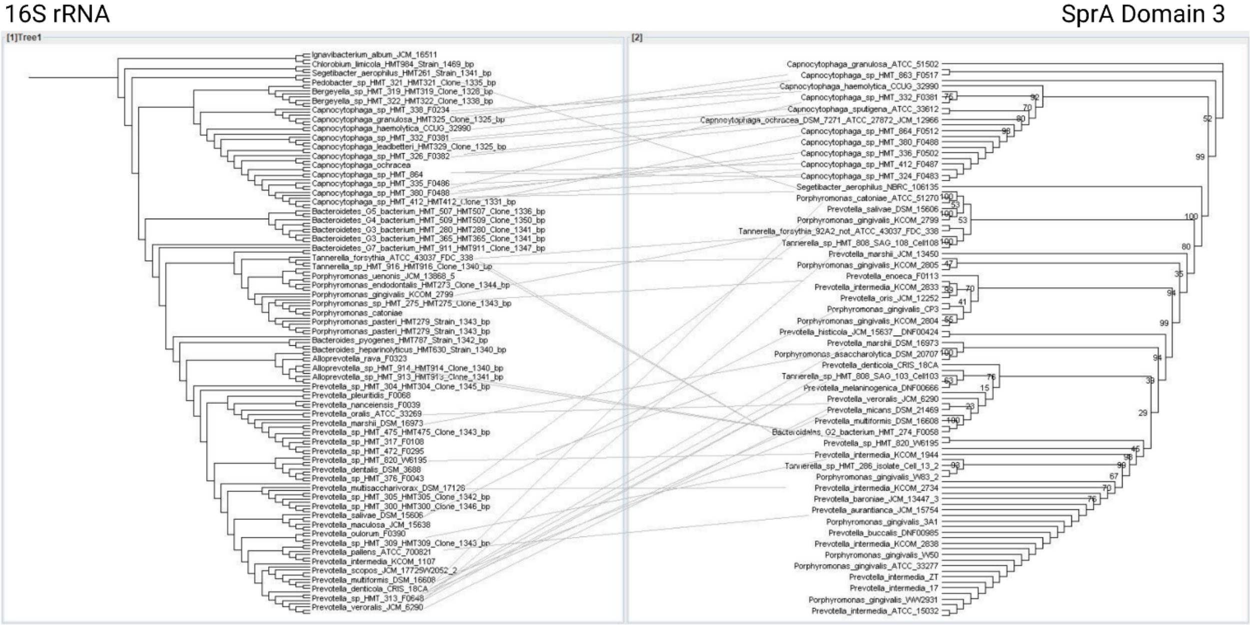

Figure S6. Tanglegram of 16S rRNA tree (Left) and the third domain of SprA (Right)

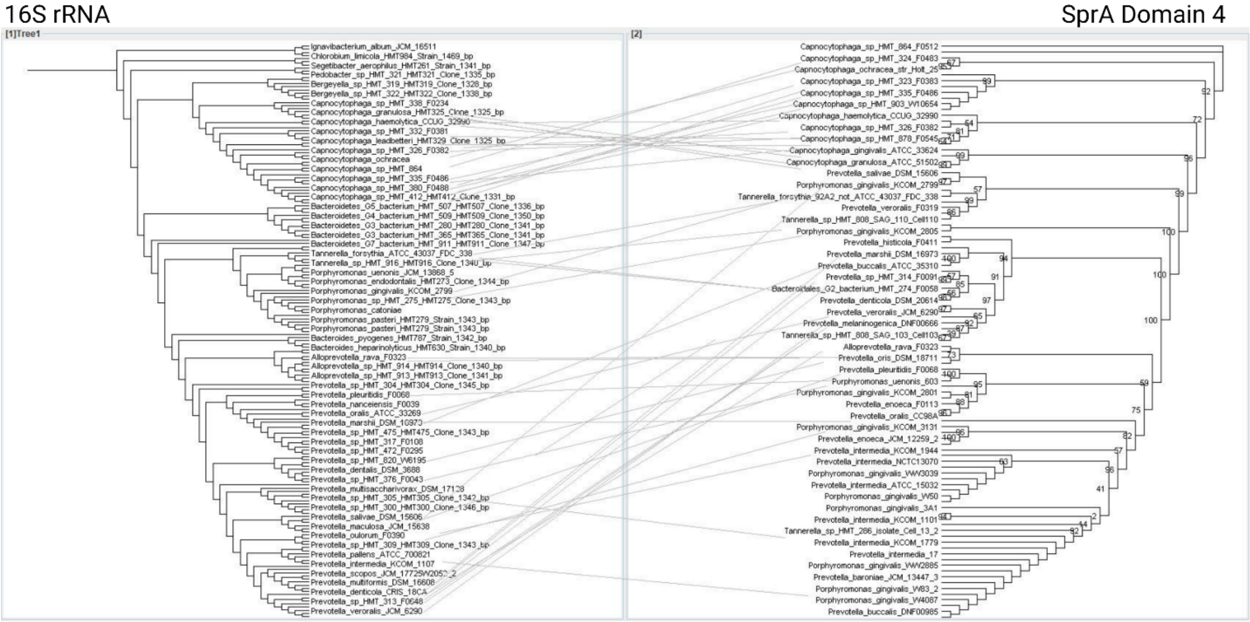

Figure S7. Tanglegram of 16S rRNA tree (Left) and the fourth domain of SprA (Right)

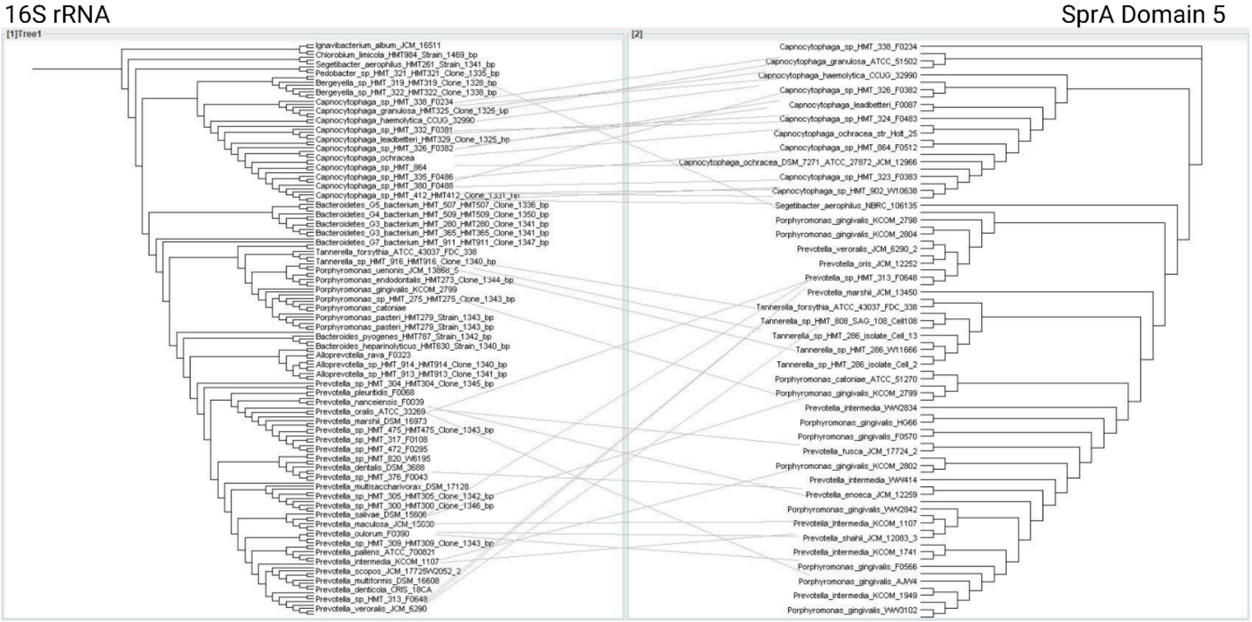

Figure S8. Tanglegram of 16S rRNA tree (Left) and the fifth domain of SprA (Right)

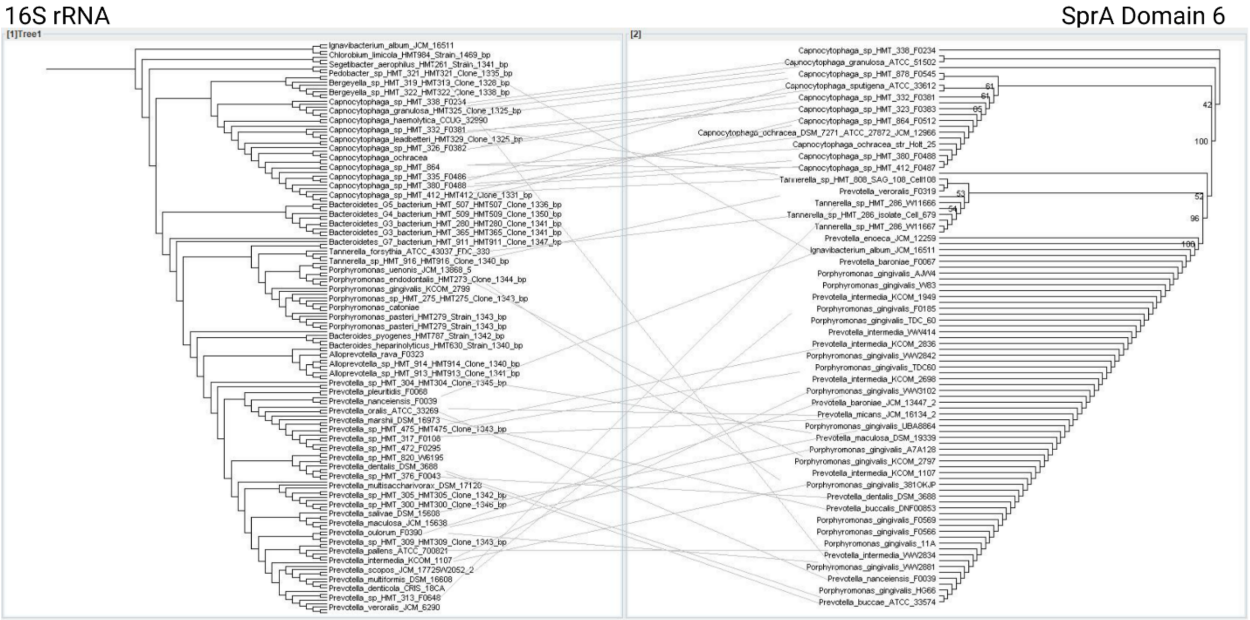

Figure S9. Tanglegram of 16S rRNA tree (Left) and the sixth domain of SprA (Right)

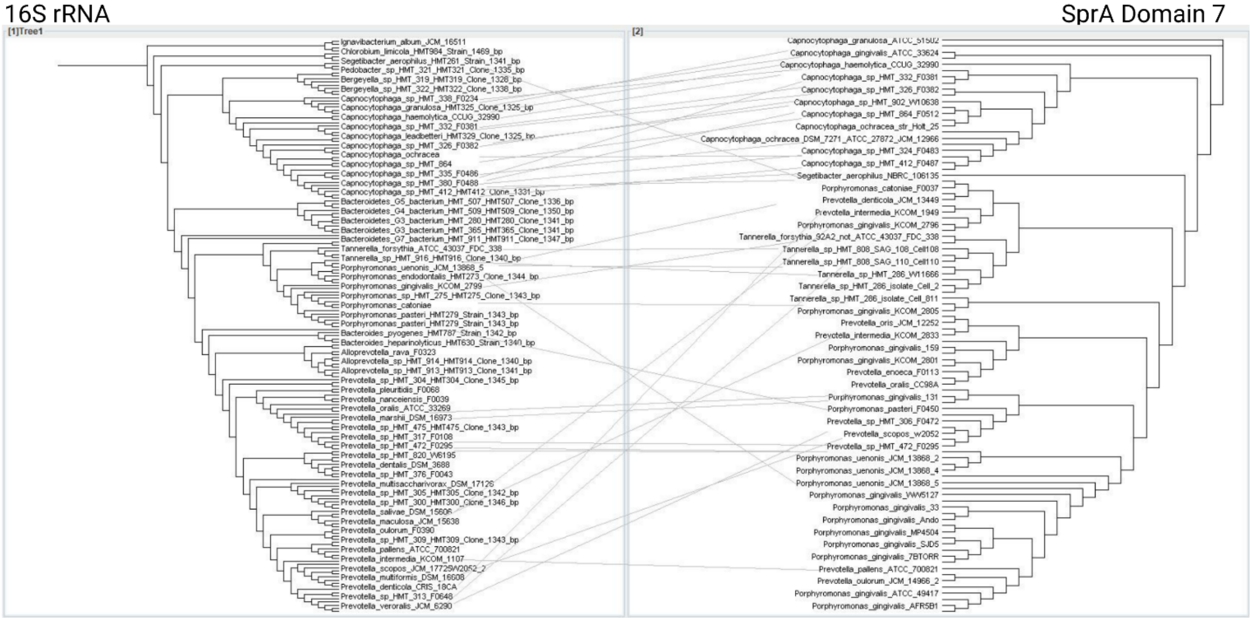

Figure S10. Tanglegram of 16S rRNA tree (Left) and the seventh domain of SprA (Right)

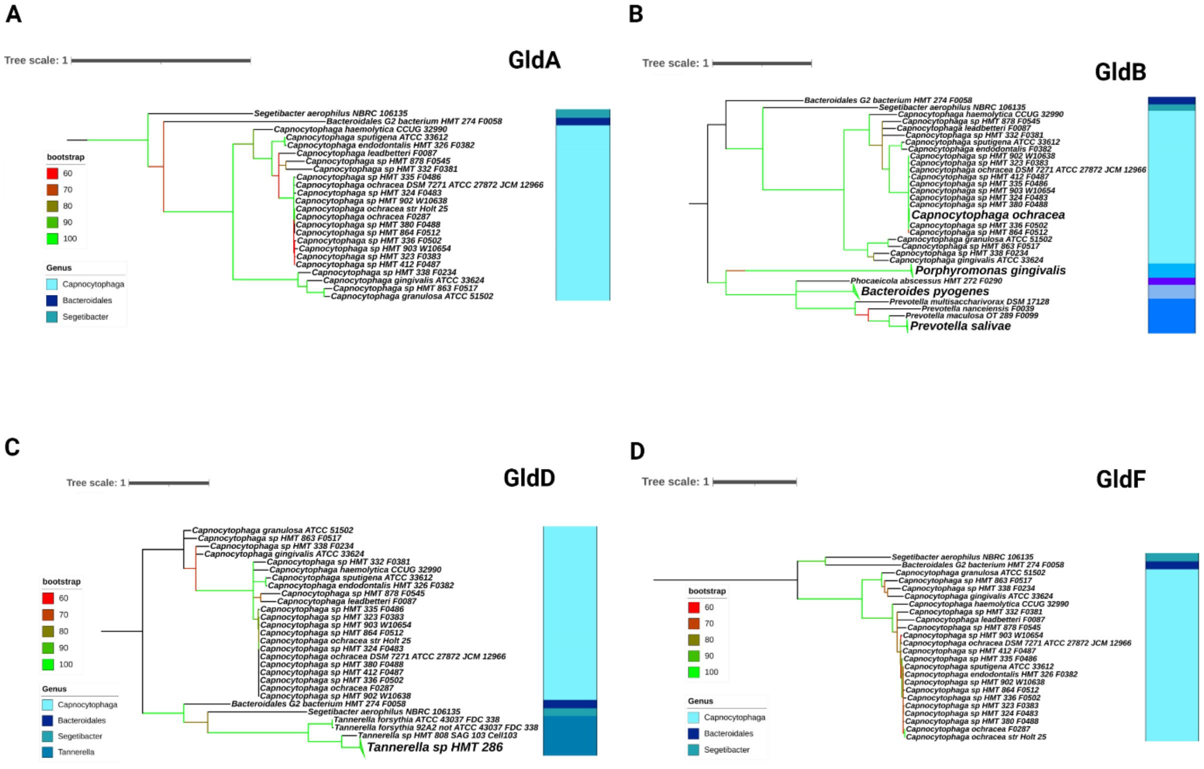

Figure S11. A. Phylogenetic tree of GldA, B. Phylogenetic tree of GldB, C. Phylogenetic tree of GldD, D. Phylogenetic tree of GldF.

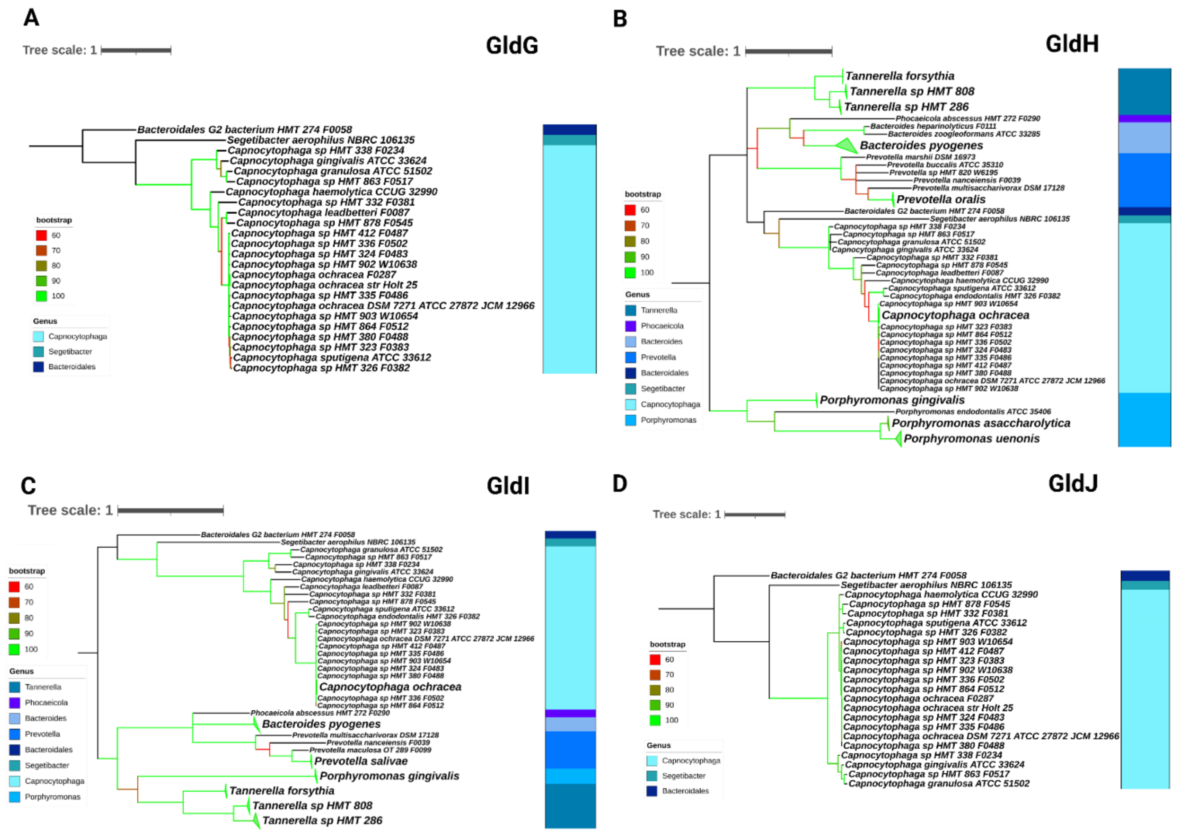

Figure S12. A. Phylogenetic tree of GldG, B. Phylogenetic tree of GldH, C. Phylogenetic tree of GldI, D. Phylogenetic tree of GldJ.

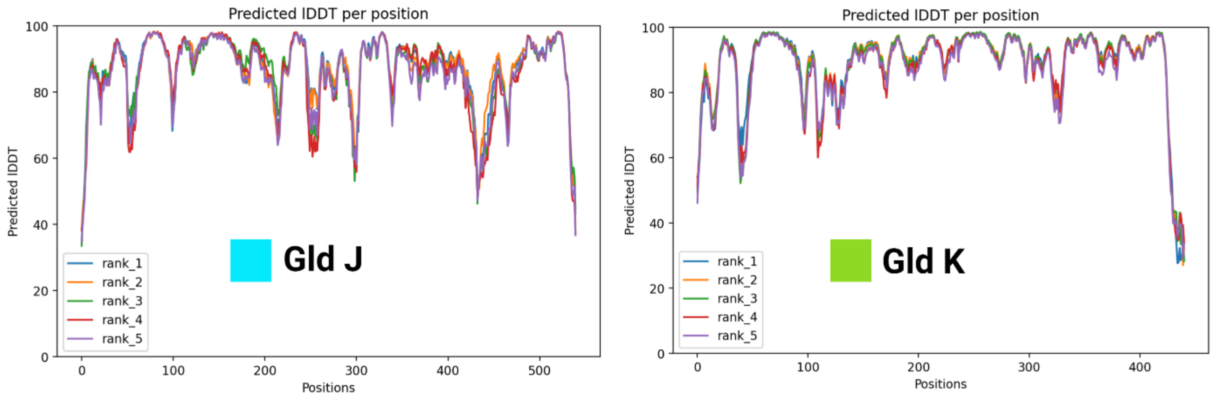

Figure S13. Graphs of the local distance difference test score vs. residue numbers for CG GldJ and CG GldK.

A

```
>Capnocytophaga_gingivalis_ATCC_33624_GldJ
MDMQSLKKPMLASLLVAAFLVGCGKKGSDKNTS
SATGWKINAKEGGFQYNTNFKEQVTGPGLVFVEG
GTFTMGKVQDDVMHDWNNTPSQQHVQSFYMDE
TEVTNKMYMEYLDWLKQVFPPEDKDGHYKNIYLG
ALPDTLVWRSPLSANETMVSEYLRHPAYAEYPVV
GVNWVQATQFSSWRTNRVNESILEKDGYIQQGSRY
QVDAESTFSTDTYLNTPENSYGRKVSEFAGKKATN
KDGNVTYAKQTEGILLPEYRLPTEVEWEYAAKSLN
GIREYNSIRGRKKYPWAGRYTRNGKRAEMGNQMA
NFKQGQGDYGGLAGWSEDKGDITTKVKTFAPNDF
GLYDMAGNVAEWVADVYRPRVDDELSDFNYYRG
NVYTKNKIGEDGKLVVVDAQSITYDTLSNGRIIARN
LPGQLAKVAVDDNETYLRYNFTTADNRNYRDGDR
VSSKEYAKLTMDRHSENIDSLTASQRANVSRTMYN
SPNQKILSDGQGGIIKVYDRSNDRTTLIDDEARVIKG
GSWRDRAYWIDPAQRRYLPQYSATDYIGFRCAMSR
VGSKAKKGKSSRG
```

B

```
>Capnocytophaga_gingivalis_ATCC_33624_GldK
MILKLKKVALIVIGYLVVSCGSGNDRGELVGVKGGQ
WKQPKPYGMTLVPGGAFVMGSSDYDKAHTEDATT
KTVTVSPFYMDDTEITNGEYRQFVNWVRDSIVRRKL
AIMAEESGQSAGGEGIGQYAFLDTDQEKLNAYDKYM
YENYGDKDNPEKRKINRKVKLIWDKEKYPDEHYVEV
MDTMYVPIEETYNGRRPLDVDKLVFKYQWMDIEAA
ARSHKKGRKDFIKEEIVKIYPDTTVWIKDFNYSYNEP
MHNDYFWHYAYSEYPVVGVTWRQAKAFCEWRTLY
KNSFQKAKKKKDVNAFRLPTEAEWEFAARGGLEGA
TYPWGGPYTVDSKACFMANFKPTRGDYAADNALY
TVEAKSYHPNGYNLYNMAGNVSEWTNTSYDSNSY
EYMSSMNPNVNDQKNKRKVIRGGSWKDVAFYTQV
ATRDYEYQDSARSYIGFRTVQSFMGVQRVDTKKGNL
TNMR
```

Figure S14. A. Amino acid sequence for GldJ. B. Amino acid sequence for GldK. Regions for structure similarity are highlighted. Each color corresponds to a separate area of similarity.

Motility type	Protein	E-Value
Flagellar	FliC	0.01
	FlgK	0.01
	FlgL	0.01
Twitching	PilT	0.01
	PilE	0.01
	PilA	0.01
Gliding	GldA	1.00E-50
	GldB	0.01
	GldD	0.01
	GldF	1.00E-31
	GldG	0.01
	GldH	1.00E-04
	GldI	0.01
	GldJ	1.00E-10
	GldK	0.01
	GldL	0.01
	GldM	0.01
	GldN	0.01
	SprA	0.01
	PorV	1.00E-10
	Plug	0.01
	PorT	0.01
	SprE	0.01

Figure S15. Table describing the E-values used for HOMD BLAST.

MAFFT on XSEDE - Parameters

accuratelyadjust_direction_	false
adjust_direction_	false
anysymbol_	false
auto_analysis_	true
datatype_	0
fmodel_	0
memSave_	0
more_memory_	false
noScore_	1
opPenaltyGroupToGroup_	1.53
outputFormat_	0
outputGuideTree_	0
outputOrder_	--inputorder
preservecase_	false
runtime_	1
usePartTree_	0
use_add_	false
use_addfrag_	false
use_addprof_	false
use_mafftthomologs_	false
use_merge_	false
use_seed_	false
which_mafft_	7490

Figure S16. Parameters set for all MAFFT files.

TrimAl on XSEDE - Parameters

out_htmlfilename_	false
print_scc_	false
print_sct_	false
print_sgc_	false
print_sgt_	false
print_sident_	false
runtime_	0.5
select_colnumbering_	false
select_complementary_	false
select_numcores_	1
specify_automated1_	false
specify_gappyout_	false
specify_noallgaps_	false
specify_nogaps_	false
specify_strict_	false
specify_strictplus_	false

Figure S17. Parameters set for all TrimAl files

FastTreeMP on XSEDE - Parameters

bionj_	false
cat_	20
close_	0.75
dist_choice_	usedefault
is_nucleotide_	Aminoacid
mlacc_	default
mllen_	false
more_memory_	false
nni_	10
nocat_	false
nome_	false
noml_	false
nosupport_	false
notop_	false
optimize_gamma_	false
protein_models_	jtt
pseudo_	true
pseudo_value_	1.0
refresh_	0.8
runtime_	10
search_speed_	default
slownni_	false
spr_	2
sprlength_	10
topm_	1.0
use_second_	true
write_log_	true
write_quoted_	false

Figure S18. Parameters set for all FastTreeMP files.

Supplementary Tables.

Table S1. Comparison table of the flagellar motor and the virulence-associated type III injectosome structural proteins.

Table S2. Summarized motility predictions of species cataloged on eHOMD. Bacterial species found to be present in a phylogenetic tree of a motility protein were marked with a green 'X'. Our motility predictions are compared with the predictions of Madin *et al*.

Table S3. Comparison table of type 4 pilus driven twitching motility proteins and type 2 secretion system proteins.

Table S4. Parameters for IQ-visualized phylogenetic tree.
